# Expression and Function of Transient Receptor Potential Ankyrin 1 Ion Channels in the Caudal Nucleus of the Solitary Tract

**DOI:** 10.3390/ijms20092065

**Published:** 2019-04-26

**Authors:** Lin Feng, Victor V. Uteshev, Louis S. Premkumar

**Affiliations:** 1 Department of Pharmacology, Southern Illinois University School of Medicine, Springfield, IL 62702, USA; lfeng@siumed.edu (L.F.); Victor.Uteshev@unthsc.edu (V.V.U.); 2 Department of Pharmacology and Neuroscience, University of North Texas Health Science Center, Fort Worth, TX 76107, USA

**Keywords:** ion channel, transient receptor potential ankyrin 1 (TRPA1), synaptic transmission, nucleus tractus solitarius (NTS)

## Abstract

The nucleus of the solitary tract (NTS) receives visceral information via the solitary tract (ST) that comprises the sensory components of the cranial nerves VII, IX and X. The Transient Receptor Potential Ankyrin 1 (TRPA1) ion channels are non-selective cation channels that are expressed primarily in pain-related sensory neurons and nerve fibers. Thus, TRPA1 expressed in the primary sensory afferents may modulate the function of second order NTS neurons. This hypothesis was tested and confirmed in the present study using acute brainstem slices and caudal NTS neurons by RT-PCR, immunostaining and patch-clamp electrophysiology. The expression of TRPA1 was detected in presynaptic locations, but not the somata of caudal NTS neurons that did not express TRPA1 mRNA or proteins. Moreover, caudal NTS neurons did not show somatodendritic responsiveness to TRPA1 agonists, while TRPA1 immunostaining was detected only in the afferent fibers. Electrophysiological recordings detected activation of presynaptic TRPA1 in glutamatergic terminals synapsing on caudal NTS neurons evidenced by the enhanced glutamatergic synaptic neurotransmission in the presence of TRPA1 agonists. The requirement of TRPA1 for modulation of spontaneous synaptic activity was confirmed using TRPA1 knockout mice where TRPA1 agonists failed to alter synaptic efficacy. Thus, this study provides the first evidence of the TRPA1-dependent modulation of the primary afferent inputs to the caudal NTS. These results suggest that the second order caudal NTS neurons act as a TRPA1-dependent interface for visceral noxious-innocuous integration at the level of the caudal brainstem.

## 1. Introduction

The brainstem nucleus of the solitary tract (NTS) is the key integrating relay in the central processing of sensory information from the thoracic and most subdiaphragmatic viscera [1,2,3]. The solitary tract (ST) is a bundle of sensory nerve fibers that extends longitudinally and bilaterally through the brainstem medulla. It comprises the sensory components of the cranial nerves VII, IX and X and relays information from both nociceptors and innocuous sensory receptors of the visceral organs and other tissues to the NTS. The ST relays information to the NTS from sensory receptors of the visceral organs and other tissues [4,5,6,7,8,9]. The NTS is a highly heterogeneous population of neurons, where seemingly indistinguishable neighboring neurons could participate in very different autonomic (e.g., gastrointestinal and cardiorespiratory reflexes) and nociceptive functions [8,10,11,12,13,14].

While glutamate is the major excitatory neurotransmitter in the brainstem, synaptic transmission at the level of the NTS can be modulated via the activation of multiple types of presynaptic ligand-gated ion channels such as transient receptor potential (TRP) channels [15,16,17,18,19,20]. The TRP ankyrin 1 (TRPA1) ion channels are nonselective cation channels highly permeable to Ca^2+^ ions. Results from animal models of visceral pain suggest that activation of TRPA1 is critical for transmission of visceral pain and may be implicated in visceral pain sensation in patients with colitis, gastric distention and inflammatory bowel disease [21,22,23,24,25,26,27].

TRPA1 may modulate neuronal and synaptic activity via diverse pathways because thermal, chemical and mechanical stimuli have been shown to activate TRPA1 in various animal models [28,29,30,31,32,33,34]. However, while TRPA1 are expressed predominantly in sensory neurons (trigeminal, superior cervical, nodose and dorsal root ganglia neurons), but not in second order neurons [30,35,36,37] and thus, not expected to be expressed in the NTS. Activation of TRPA1 in nerve terminals that synapse onto NTS neurons may have prominent effects on neuronal function and synaptic transmission within the NTS. This hypothesis is tested in the present study using acute brainstem slices of caudal NTS neurons. Our findings suggest that the second order caudal NTS neurons act as a TRPA1-dependent interface for visceral noxious-innocuous integration at the level of the caudal brainstem.

## 2. Results

### 2.1. Presynaptic Expression of TRPA1 in the Caudal NTS

**Figure 1:**
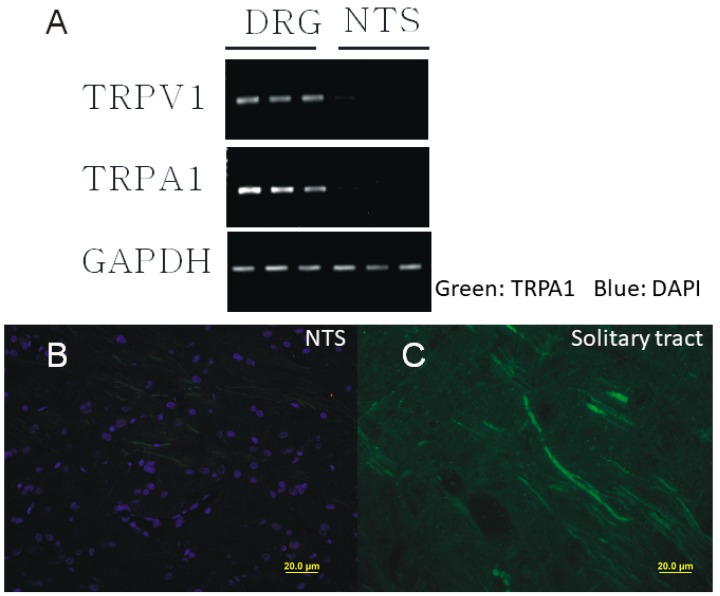
The lack of expression of TRPA1 in the nucleus of the solitary tract (NTS). (**A**) Expression of TRPA1 mRNA in sensory neurons but not in the NTS. (**B**,**C**) Immunostaining of TRPA1 (green), DAPI (blue) in the NTS. The TRPA1 staining was detected abundantly on the solitary tract, but not within the NTS. DRG: dorsal root ganglion.

**Figure 2:**
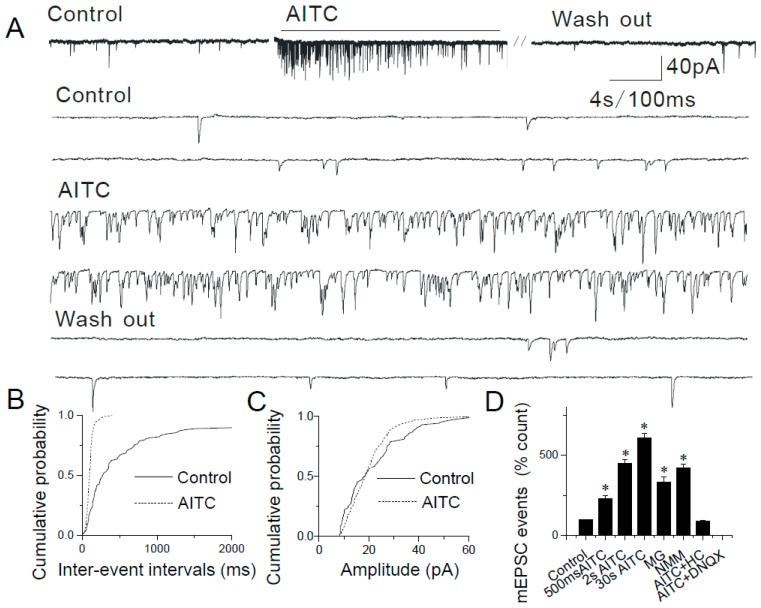
Modulation of synaptic transmission in the NTS by allyl isothiocyanate (AITC). (**A**) Application of AITC (200 µM) increases the frequency of miniature excitatory synaptic activity (mEPSCs) in a reversible manner. The synaptic events are shown in higher time resolution below. (**B**) Cumulative probability plot showing decreased inter-event intervals representing increased frequency of mEPSCs (*p* < 0.0001, KS test). (**C**) The increase in frequency is not accompanied by a change in the amplitude. (**D**) Summary graph showing AITC-mediated increases in the frequency of mEPSCs in a dose-dependent manner (* *p* < 0.05). Furthermore, the increase in AITC -induced synaptic events are blocked by 300 μM HC030031 (*n* = 5, * *p* < 0.05). The asterisk (*) represents *p* < 0.05 as compared to control.

**Figure 3:**
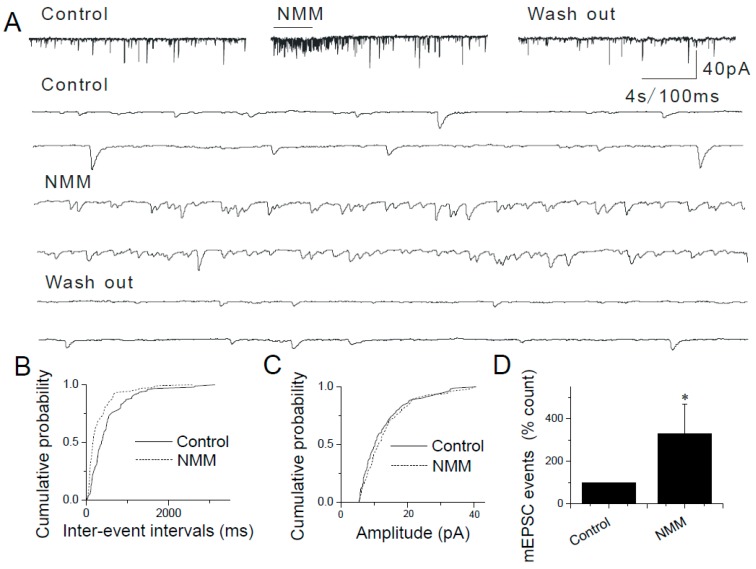
Modulation of synaptic transmission in the NTS by N-methylmaleimide (NMM). (**A**) Application of NMM (100 µM) increases the frequency of mEPSCs in a reversible manner. The synaptic events are shown in higher time resolution below. (**B**) Cumulative probability plot showing decreased inter-event intervals representing increased frequency of mEPSCs (*p* < 0.0001, KS test). (**C**) The increase in frequency is not accompanied by a change in the amplitude. (**D**) Summary graphs showing that the NMM-mediated increase in mEPSCs (*n* = 8, * *p* < 0.05). The asterisk (*) represents *p* < 0.05 as compared to control.

**Figure 4:**
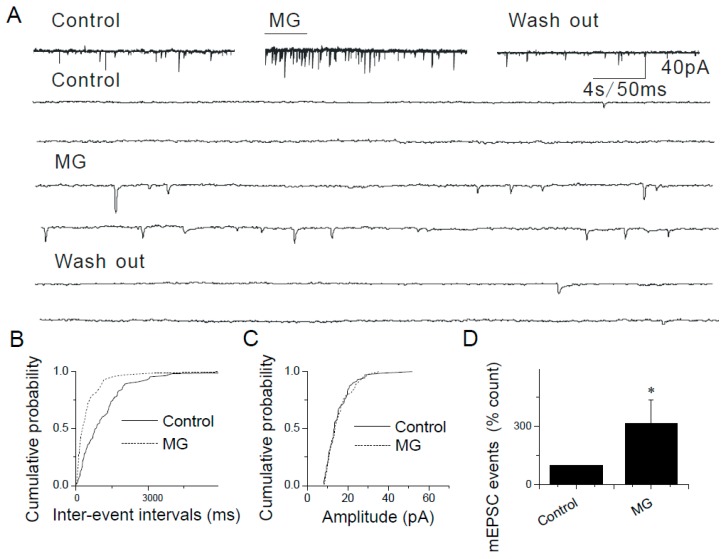
Modulation of synaptic transmission in the NTS by methylglyoxal (MG). (**A**) Application of MG (50 µM) increases the frequency of mEPSCs in a reversible manner. The synaptic events are shown in higher time resolution below. (**B**) Cumulative probability plot showing decreased interevent intervals representing increased frequency of mEPSCs (*p* < 0.0001, KS test). (**C**) The increase in frequency is not accompanied by a change in the amplitude. (**D**) Summary graphs showing that MG-mediated increase in mEPSCs (*n* = 7, * *p* < 0.05). The asterisk (*) represents *p* < 0.05 as compared to control.

The existing literature indicates that sensory TRP channels are predominantly expressed in peripheral neurons and their expression in central neurons is limited [38,39]. To determine whether this rule applies to TRPA1 in the NTS, we used RT-PCR and immunohistochemistry. The TRPA1 mRNA was not detected in caudal NTS neurons while its presence was clearly detected in DRG neurons that served as a positive control (Figure 1A). Immunofluorescent staining then revealed that TRPA1 in the caudal NTS are selectively expressed only in nerve fibers i.e., pre-synaptically (Figure 1B,C), which is consistent with the expression of TRPA1 in the solitary tract fibers, but not the somatic expression in the second order caudal NTS neurons. However, these results alone do not rule out the expression of TRPA1 in non-solitary tract terminals and will be further confirmed in conjunction with our electrophysiological data (see Figure 2, Figure 3 and Figure 4).

### 2.2. Modulation of Synaptic Transmission by AITC in the Caudal NTS

To determine the functional characteristics of TRPA1 expressed in pre-synaptic terminals that synapse onto caudal NTS neurons, we used acute horizontal brainstem slices and patch-clamp electrophysiology. In voltage-clamp experiments, visualized caudal NTS neurons were held at −60 mV and synaptic currents were recorded at various experimental conditions. The expression of functional TRPA1 was confirmed using TRPA1 agonist, allyl isothiocyanate (i.e., AITC). Focal pressure puffs of AITC applied to recording caudal NTS neurons robustly increased the frequency of spontaneous excitatory synaptic currents (sEPSCs) (data not shown). To determine the effects of AITC on miniature excitatory synaptic activity (mEPSCs), patch-clamp recordings were conducted in the presence of 1 μM tetrodotoxin (TTX) to inhibit voltage-gated Na^+^ channels and prevent action potential-dependent synaptic events. In these experiments, application of AITC (200 µM) significantly increased the frequency of mEPSCs in a pulse duration-/concentration-dependent manner (Figure 2).

The increase in mEPSC frequency is expressed as a percentage of control (i.e., no drugs applied): 500 ms, 230.25 ± 20.41% (*n* = 8, *p* < 0.05): 2 s, 450.63 ± 22.74% (*n* = 13, *p* < 0.05); 30 s, 610.79 ± 30.81% (*n* = 9, *p* < 0.05) (Figure 2A,B,D). The means mEPSC amplitude did not significantly change (control, 19.53 ± 2.53 pA, *n* = 5; after AITC, 21.2 ± 3.09 pA, *n* = 12) (Figure 2C). The increase in the mEPSC frequency, but not the amplitude suggests that AITC acts only pre-synaptically, which is consistent with the pre-synaptic expression of TRPA1 (Figure 1). These potentiating effects of AITC were observed in ~40% (*n* = 143/358) of tested caudal NTS neurons. These results suggest that only neurons that received primary sensory afferent input responded by increasing the frequency of mEPSCs following AITC application.

mEPSCs were completely blocked by 6,7-Dinitroquinoxaline-2,3-dione (i.e., DNQX; 16 μM), a selective antagonist of AMPA receptors (Figure 2D). The involvement of TRPA1 was confirmed using HC030031 (i.e., HC), a TRPA1 selective antagonist. HC (300 µM) abolished the effects of AITC on mEPSC frequency without affecting the background synaptic activity (Figure 2D).

Similar results were obtained in experiments where two other TRPA1 agonists (i.e., N-methylmaleimide (i.e., NMM), an oxidizing agent that forms a covalent bond with TRPA1 and methylglyoxal (i.e., MG), a reactive molecule and an endogenous TRPA1 agonist, produced during hyperglycemia) were used. Pressure puffs of NMM or MG increased the frequency, but not amplitude of mEPSCs in caudal NTS neurons (Figure 3 and Figure 4): 100 µM NMM (421.61 ± 24.09%, *n* = 8, *p* < 0.05; Figure 3B,D) and 50 µM MG (334.76 ± 30.62% *n* = 7, *p* < 0.05; Figure 4B,D).

By contrast, the somatodendritic responsiveness between NTS neurons to AITC, NMM and MG has not been detected in this study. Together with the molecular biological results (Figure 1), these data (Figure 2, Figure 3 and Figure 4) support a strictly pre-synaptic expression of TRPA1 either in the primary afferent (solitary) terminals and/or pre-synaptic glutamatergic terminals of second- or higher-order NTS neurons.

### 2.3. Changes in mEPSCs in Response to Continuous and Repeated Application of AITC

TRPA1 agonists AITC, NMM and MG have been shown to activate the channel by covalent modification of cysteine and lysine residues [34,40]. Although covalent modification is expected to be an irreversible process, within the time course of electrophysiological experiments, NMM and AITC activate TRPA1 in a reversible manner [34,41]. We found that brief (2–10 s) puffs of AITC (200 µM), NMM (100 µM) or MG (50 µM) induced responses that were readily reversible (Figure 2, Figure 3 and Figure 4) and a continuous application of AITC decreased the frequency of mEPSCs over time (Figure 2A).

**Figure 5:**
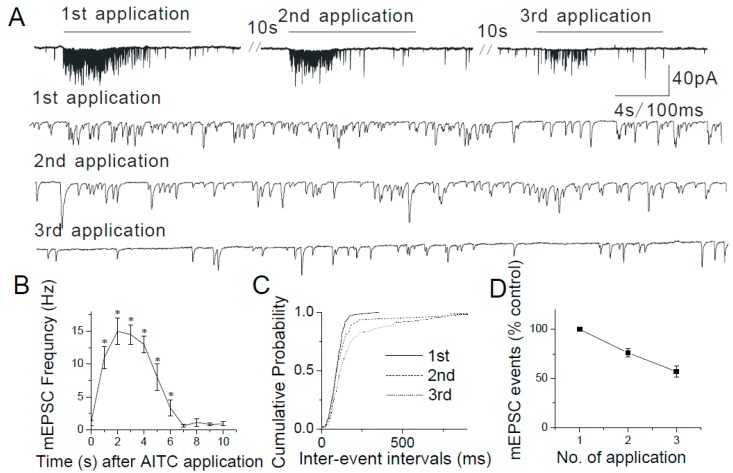
The tachyphylaxis of TRPA1. (**A**) AITC (200 µM) causes enhancement of mEPSCs progressively decreases with repeated AITC application. Synaptic currents are shown in a higher time resolution below. (**B**) Summary graph representing a progressive decrease in the number of mEPSC events immediately following AITC application. (**C**) Cumulative probability plots showing decreased mEPSC frequency as indicated by a progressive increase in the inter-event intervals with each subsequent AITC application. (**D**) Summary graph representing a progressive decline in mEPSC frequency with repeated AITC application. A significant decrease in frequency is observed only between the first and third AITC application (first application, *n* = 5; third application, *n* = 5, *p* < 0.05) (first application, 357.53 ± 135.2%, *n* = 5; second application, 271.64 ± 122.57%, *n* = 5; third application, 203.85 ± 73.50% of control, *n* = 6). The asterisk (*) represents *p* < 0.05 as compared to control.

In a separate experiment, changes in mEPSC frequency were analyzed with continuous application of AITC (Figure 5A,B). The AITC-mediated facilitation of mEPSCs showed a gradual decrease with time (Figure 5B). A persistent depolarization of presynaptic terminal and/or Ca^2+^-dependent decrease in TRPA1 activity and/or desensitization of TRPA1 may be responsible for the observed run-down of synaptic activity.

To determine whether AITC causes tachyphylaxis, AITC (2 s duration) was repeatedly applied to the recorded caudal NTS neurons every 10 s separated by a washout. Successive applications of AITC (200 µM) gradually decreased the mEPSC frequency (first application, 657.53 ± 135.2%, *n* = 5; second application, 487.64 ± 122.57%, *n* = 5; third application, 374.85 ± 73.50% of control, *n* = 6) (Figure 5A,C,D). These data support that TRPA1-mediated transmitter release is decreased with repeated application of AITC. Thus, in all experiments with multiple applications of agonists, at least 3 min of washout was given after each agonist application to avoid desensitization.

### 2.4. The Lack of Sensitization of Presynaptic TRPA1 by PDBu in the Caudal NTS

**Figure 6:**
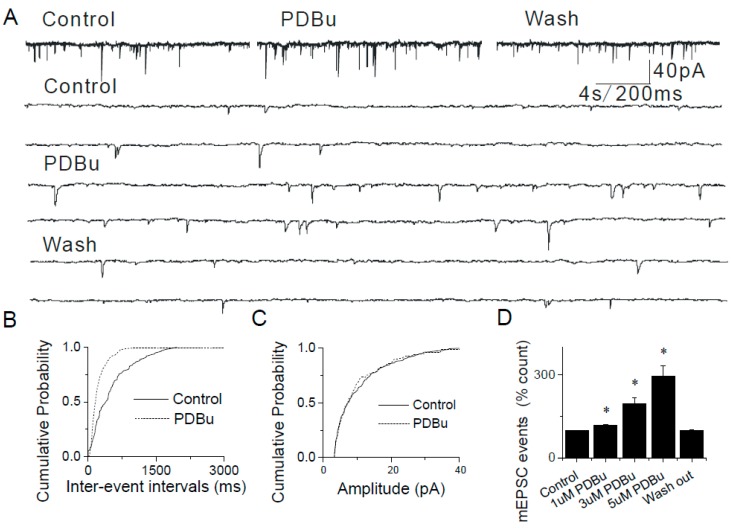
Modulation of synaptic transmission in the NTS by phorbol-12,13-dibutyrate (PDBu). (**A**) PDBu increases the frequency of mEPSCs in a reversible manner. Synaptic currents are shown in higher time resolution below. (**B**,**C**) Cumulative probability graphs showing enhanced frequency of mEPSCs (*p* < 0.001,KS test) in response to PDBu without change in their amplitudes. (**D**) Summary graphs showing that PDBu-mediated increase in mEPSCs (1 µM, 117.84 ± 19.66%, *n* = 6; 3 µM, 195.76 ± 30.15%, *n* = 5, * *p* < 0.01; 5 μM, 295.13 ± 38.57%, *n* = 6, * *p* < 0.01). The asterisk (*) represents *p* < 0.05 as compared to control.

Application of phorbol-12,13-dibutyrate (PDBu, 1–5 μM, a PKC activator) alone induced a dose-dependent increase in the frequency, but not amplitude of mEPSCs in caudal NTS neurons (Figure 6A,B,D). The effects of PDBu on mEPSC frequency were significant: 1 µM, 117.84 ± 19.66%, *n* = 6; 3 µM, 195.76 ± 22.15%, *n* = 5, *p* < 0.01; 5 μM, 295.13 ± 28.57%, *n* = 6, *p* < 0.01 (Figure 6D), whereas the mEPSC amplitudes remained unaltered (Figure 6C), supporting a presynaptic mechanism of action of PDBu [42,43,44,45].

**Figure 7:**
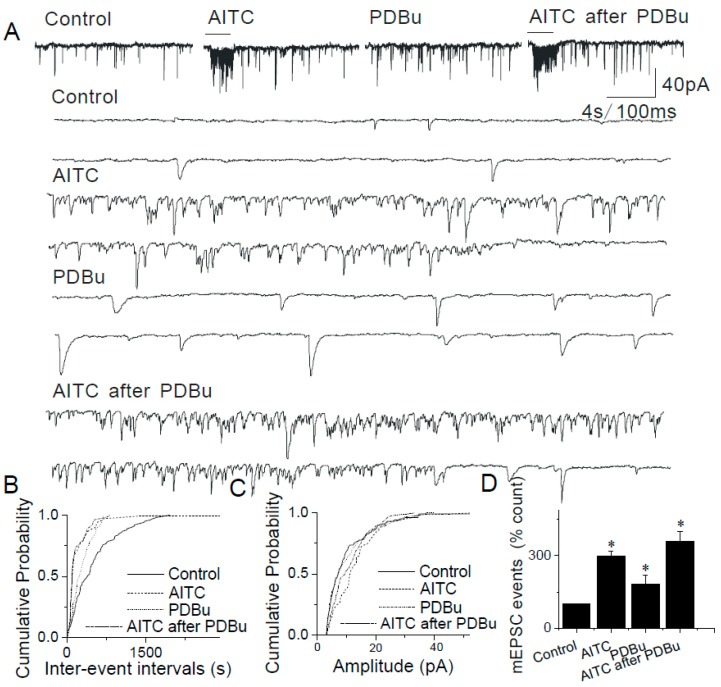
Interaction between AITC and PDBu in the NTS. (**A**) Application of AITC (200 μM) increases the frequency of mEPSCs and this action is enhanced by incubation in 3 µM PDBu. Synaptic events are shown at a higher time resolution below. (**B**) The cumulative probability plots show an AITC-mediated increase in the frequency of synaptic events. These effects are further significantly enhanced by PDBu. (**C**) The increase in frequency of events is not accompanied by a change in the amplitude. (**D**) Summary graphs showing AITC-induced an increase in mEPSC frequency (*n* = 9) and potentiation by PDBu (*n* = 8). The asterisk (*) represents *p* < 0.05 as compared to control.

Following application of PDBu (3 μM) to the ACSF, AITC increased the mEPSC frequency, but not amplitude (AITC alone, 298.51 ± 21.86%; AITC + PDBu, 358.69 ± 42.58% (*n* = 9, *p* > 0.05)) (Figure 7A,B,D). The effects of PDBu and AITC on mEPSC frequency appear to be additive, not synergistic suggesting that PDBu and AITC employ two different and independent mechanisms of action in presyanptic terminals of caudal NTS neurons.

### 2.5. The Lack of Modulatory Effects of AITC on Inhibitory Synapses in the Caudal NTS

**Figure 8:**
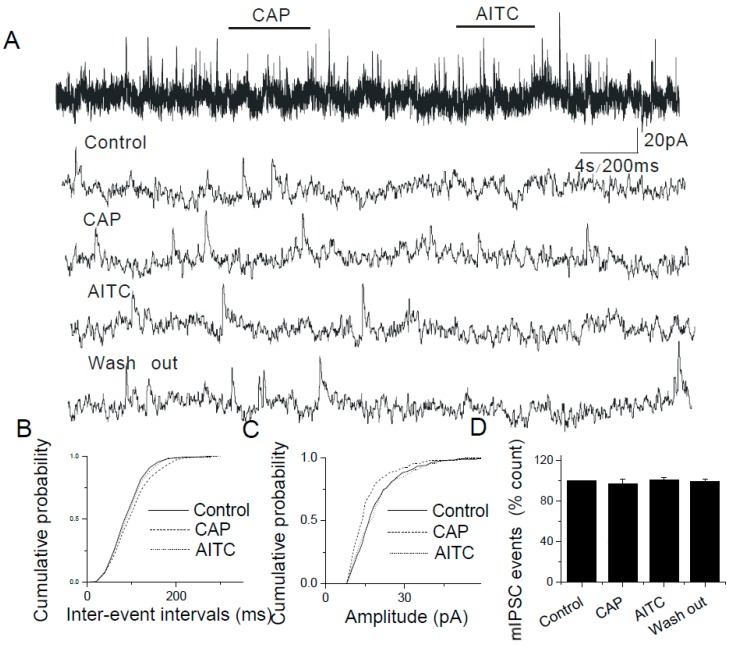
AITC does not alter inhibitory synaptic transmission in the NTS. (**A**) Administration of AITC (500 µM) or capsaicin (i.e., CAP; 200 nM) did not have any effect on the frequency of mIPSCs (top trace). Bottom four traces: the same synaptic events are shown at a higher time resolution. (**B**,**C**) The cumulative probability plots show no significant changes (*p* < 0.0001, KS test) in the distributions of inter-event intervals (**B**) and amplitudes (**C**) of mIPSCs. (**D**) A summary graph illustrating a lack of effects of AITC on the mIPSC frequency (*n* = 10).

In experiments where AMPA and NMDA receptors were blocked with DNQX (16 μM) and 2-amino-5-phosphonovaleric acid (APV, 20 μM), respectively, added to ACSF, pressure application of AITC (500 µM) failed to alter either the frequency or the amplitude of mIPSCs (AITC, 97.86 ± 8.64%, *n* = 10, Figure 8). Therefore, AITC does not affect the release of inhibitory synaptic neurotransmitters.

### 2.6. Modulation of Evoked EPSCs by AITC in the Caudal NTS

**Figure 9:**
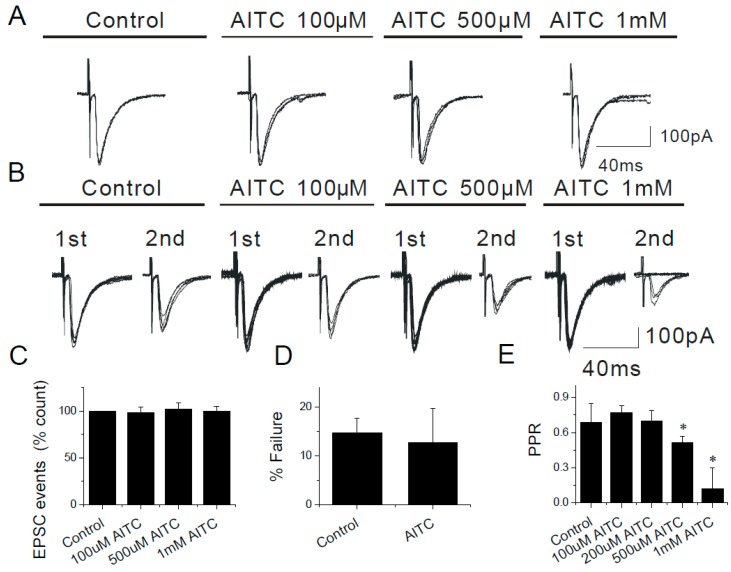
AITC-induced effects on solitary tract-evoked synaptic responses in horizontal brainstem slices. (**A**,**B**) Representative traces of solitary tract-stimulated EPSCs (ST-EPSCs) showing seven overlapping sweeps. (**A**,**C**) AITC does not have any significant effect on the amplitude of the ST-EPSCs (*n* = 14). (**B**,**E**) AITC depressed the paired pulse ratio (PPR, EPSC2/EPSC1) in a concentration dependent manner. (**D**) AITC does not show any significant effect on the failure rate (% failures = (number of failures/total stimulations) × 100%) in NTS neurons (*n* = 11). The asterisk (*) represents *p* < 0.05 as compared to control.

AITC significantly modulated evoked EPSCs generated by electrical stimulation of the solitary tract (50–400 µs, pulse duration; 100–500 µA, pulse intensity; 60 s inter-pulse interval). The average amplitude of EPSCs was 131.86 pA ± 41.29 (*n* = 14) (ranged between 40.00 pA to 396.19 pA) and AITC (100 µM) failed to alter the EPSC amplitude or kinetics (*p* > 0.05; *n* = 14, Figure 9A,C). In experiments where a paired-pulse protocol was employed, AITC significantly depressed the paired-pulse ratio (PPR) in a concentration-dependent manner (control, 0.6877 ± 0.163, *n* = 12; 100 µM, 0.770 ± 0.060, *n* = 10; 200 µM, 0.7 ± 0.086, *n* = 4; 500 µM, 0.517 ± 0.051, *n* = 7, *p* < 0.05; 1 mM, 0.117 ± 0.179, *n* = 8, *p* < 0.05; Figure 9B,D). It is interesting to note that stimulation of TRPV1 significantly inhibited the evoked responses as reported by Peters et al., 2010 [46]. The differential modulation of synaptic transmission by TRPA1 and TRPV1 is intriguing. These results are consistent with the expression of TRPA1 in the primary afferent solitary tract terminals in the caudal NTS.

### 2.7. Data from TRPA1 Knockout Mice Support the Modulatory Role of TRPA1 in the Caudal NTS

**Figure 10:**
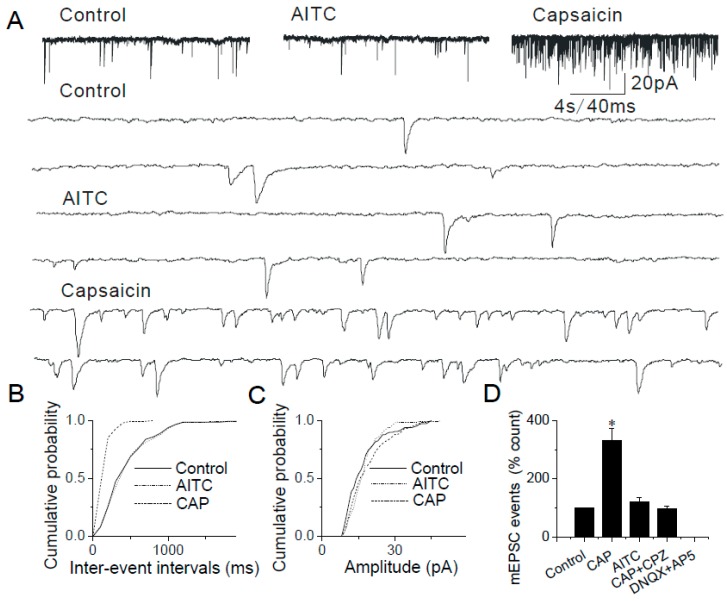
AITC does not induce enhancement of synaptic transmission in the TRPA1 knockout mice. (**A**) Application of AITC (200 µM) does not increases the frequency of mEPSC in the TRPA1 knockout mice (105.21 ± 12.23%, *n* = 15, *p* > 0.05); whereas, capsaicin increase the frequency of mEPSC significantly (309 ± 42%, *n* = 7, *p* < 0.05); The synaptic events are shown in higher time resolution below. (**B**) Cumulative probability plot showing decreased interevent intervals representing increased frequency of mEPSCs mediated by capsaicin (i.e., CAP; *p* < 0.0001, KS test), but no change of interevent intervals mediated by AITC. (**C**) There was no change in the amplitude in all three groups. (**D**) Summary graph showing only capsaicin-mediated increases but no AITC mediated changes in the frequency of mEPSCs (* *p* < 0.05). The asterisk (*) represents *p* < 0.05 as compared to control.

TRPA1 knockout mice were used to further elucidate the modulatory role of presynaptic TRPA1 in the caudal NTS. In experiments using brainstem slices from TRPA1 knockout mice, AITC (200 μM) was ineffective in increasing mEPSC frequency (as a percent of control): 105.21 ± 12.23% (*n* = 15, Figure 10A,B,D; vs. effects of AITC in caudal NTS neurons obtained from wild-type mice: 450.63 ± 22.74% (*n* = 13, *p* < 0.05) Figure 2A,B,D). In the same experiments, TRPV1 agonist, capsaicin (100 nM) significantly increased the frequency of mEPSCs (309.88 ± 42.06%, *n* = 7, Figure 10A,B,D). These experiments further confirmed that the observed effects of AITC were mediated by TRPA1.

## 3. Discussion

In this study, the molecular biological and electrophysiological techniques were used to demonstrate TRPA1-mediated modulation of synaptic transmission at the NTS and the expression of TRPA1 in caudal NTS is strictly presynaptic. Together with the existing literature, these results support the exclusive expression of TRPA1 in the first order sensory neurons and indicate that the expression of TRPA1 in the caudal NTS is restricted to the primary afferent solitary tract terminals [30,35,36,47]. Activation of presynaptic TRPA1 by selective agonists was detected by its facilitating and modulatory effects on spontaneous and evoked glutamatergic synaptic transmission, respectively, recorded electrophysiologically in acute horizontal brainstem slices; while the specificity of TRPA1 in triggering synaptic glutamate release was confirmed in TRPA1 knockout mice. The effects of TRPA1 activation on glutamatergic synaptic transmission in the caudal NTS are consistent with those observed previously in other central sensory nuclei: the substantia gelatinosa and the caudal spinal trigeminal nucleus [30,48,49,50,51,52,53]. Agonists of TRPA1 (AITC) and TRPV1 (capsaicin; i.e., CAP) caused paired pulse depression. However, AITC did not depress evoked EPSC amplitude, while capsaicin did depress the evoked EPSC amplitude as described previously [45]. This observation is curious because TRPA1 and TRPV1 channels are often co-expressed in the same subset of neurons [30] and their effects on synaptic neurotransmitter release are expected to be comparable. Differences in the sensitivity of presynaptic terminals to AITC and capsaicin, as well as the exact channel distribution within presynaptic terminals may be responsible for the apparent dichotomy of TRPA1- and TRPV1-mediated evoked presynaptic responses, respectively.

The functional role of TRPA1 in sensory signaling is suggested by the expression of these channels in subsets of primary sensory neurons in the trigeminal, jugular, geniculate, nodose and dorsal root ganglia [43,54,55,56]. The TRPA1 channels act as primary sensors for thermal (cold), mechanical and inflammatory noxious stimuli generated by environmental and endogenous agents [30,31,32,43,57,58,59,60,61,62]. Because TRPA1 channels are critical for transmission of visceral and inflammatory pain [21,22,23,24,25,26,27], the expression of functional presynaptic TRPA1 in primary afferents within the caudal NTS suggests a potential for TRPA1-dependent central component of nociception and its sensitization by TRPA1 agents at the level of the caudal brainstem.

Visceral pain is a common symptom of functional gastrointestinal disorder such as irritable bowel syndrome, ulcerative colitis and dyspepsia. As visceral structures are highly sensitive to distention, ischemia and inflammation, the features of chronic visceral pain are inflammatory and mechanical hyperalgesia and allodynia. Adopting strategies to reduce inflammatory and mechanosensory transduction may be particularly useful in relieving visceral pain [63,64,65,66]. The role of TRPA1 in gastrointestinal inflammatory disorders is becoming increasingly important as TRPA1 up-regulation has been confirmed in several disease model systems. Thus, TRPA1 may represent a useful target in treatments of chronic visceral pain. In fact, several TRPA1 antagonists have already entered clinical trials including one in phase II clinical trial [66].

A potential role of protein kinase C (PKC) in modulating TRPA1-mediated synaptic transmission was examined using PDBu, a PKC activator. The PKC-mediated phosphorylation plays an important role in modulation of synaptic neurotransmission [40,43,44,45,67,68]. For example, PDBu substantially increased the amplitude of TRPV1-mediated currents but had no effect on TRPA1-mediated currents in dorsal root ganglia neurons [41,69]. The direct PDBu-mediated facilitation of the frequency of synaptic events was observed in all neurons tested in this study, even in those cases where AITC failed to facilitate synaptic release. However, the effects of AITC and PDBu were simply additive (i.e., not synergistic) suggesting that AITC and PDBu likely employ independent pathways and thus, the TRPA1-dependent machinery is unlikely to involve PKC-mediated phosphorylation. This finding may reflect the nature of agonists used. AITC and NMM activate TRPA1 by covalent modification of cysteine residues. As a result, the affinity of these ligands for TRPA1 binding site(s) may not be altered by phosphorylation. In that event, the TRPA1 activity may still be susceptible to modulation by non-covalent modifying agonists and physical inputs, such as thermal (cold) and mechanical stimuli.

Taken together, the results of this study provide the first evidence of the TRPA1-dependent modulation of the primary afferent inputs to the caudal NTS and suggest that second order caudal NTS neurons serve as a TRPA1-dependent interface for visceral noxious-innocuous integration at the level of the caudal brainstem. Thus, TRPA1 may represent a useful target in treatments of chronic visceral and inflammatory pain.

## 4. Materials and Methods

### 4.1. Animals

Young adult male Sprague-Dawley rats (P30-50) and TRPA1 knockout mice (Jackson Laboratories, Bar Harbor, ME, USA) were used in accordance with the Guide for the Care and Use of Laboratory Animals (NIH 865-23, Bethesda, MD, USA), and was approved by the Animal Care and Use Committee of Southern Illinois University (A3209-01; Approval date: 7/25/2013).

### 4.2. Preparation of Brainstem Slices

Brains were removed and placed in an ice-cold oxygenated solution of the following composition (in mM): sucrose 250, KCl 1.5, NaH_2_PO_4_ 2.23, MgCl_2_ 5, CaCl_2_ 0.5, NaHCO_3_ 26, glucose 10 (pH 7.4) and bubbled with carbogen (95% O_2_ and 5% CO_2_). The brainstem was isolated and transferred to the cutting chamber of Vibratome-1000+ slicer (Leica Microsystems, Wetzlar, Germany) where horizontal brainstem slices (250–300 μm thickness) containing the NTS were prepared. The slices were then transferred to a storage chamber and incubated at 30 °C for ~60 min in an oxygenated artificial cerebrospinal fluid (ACSF) of the following composition (in mM): NaCl 125, KCl 1.5, NaH_2_PO_4_ 2.23, MgCl_2_ 1, CaCl_2_ 2, NaHCO_3_ 26, glucose 10 (pH 7.4). Slices were then stored in the identical oxygenated ACSF at 24 °C for up to 8 h.

### 4.3. Electrophysiological Patch-Clamp Recordings

For patch-clamp recordings, brainstem slices were placed in the recording chamber and perfused with oxygenated ACSF at a rate of 1 mL/min using a 2232 Microperpex S peristaltic pump (LK.B, Upsalla, Sweden). Slices were secured using a nylon mesh attached to platinum ring insert. Whole-cell recordings were conducted at room temperature. The patch electrode solution contained (in mM): K-gluconate 140, NaCl 1, MgCl_2_ 2, Mg-ATP 2, Na-GTP 0.3, HEPES 10, KOH 0.42 (pH 7.4). Membrane voltages were not corrected for the liquid junction potential which was calculated using software available through pCamp: VLJ = 16.2 mV. The electrophysiological data were recorded using MultiClamp-700B patch-clamp amplifier (Molecular Devices, Sunyvale, CA, USA). The seal resistance was >2 GΩ. The access resistance was <30 MΩ and was not compensated. Patches with access resistances >30 MΩ were corrected by applying additional negative suction or discarded. Input resistance and series resistance were measured every five minutes and cells showing greater that 20% change in series resistance were not included in analysis. Data were sampled at 10–20 kHz and filtered at 5 kHz. The final drug concentration in the bath was calculated based on the known concentration of the stock solution and adjustable rates of pumps [70]. A picospritzer (Parker Hannifin Instrumentation, Cleveland, OH, USA) was used for agonist applications via pipettes (4–7 MΩ) identical to those used for patch clamp recordings. Agonist application was standardized by positioning the tip of the application pipette 15 μm from the recorded neuron. This distance was calibrated and marked on the TV monitor and used for visualization of neurons while patching. Off-line data analysis was done with the program Clampfit 9 (Molecular Devices, Sunnyvale, CA, USA). To obtain evoked EPSCs, a Grass Stimulator (S88) with stimulus isolation unit with constant current output (Grass Instrument, Quincy, MA, USA) was used to stimulate a concentric bipolar electrode (Rhodes Medical Instruments, Tujunga, CA, USA) placed on the solitary tract. The following parameters of electrical stimulation were used: stimulus duration, 50–400 µs; stimulus intensity,100–500 µA; inter-stimulus interval, 60 s.

### 4.4. Immunohistochemistry and Peptide Absorption Studies

Rats were anesthetized by intraperitoneal (i.p.) injections of ketamine (85 mg/kg) and xylazine (10 mg/kg). The anesthetized animals were perfused transcardially with 4% paraformaldehyde. The brainstem was harvested and stored in the phosphate buffer saline (PBS, pH7.4) containing 30% sucrose for at least 24 h. Then the tissue was frozen in powdered dry ice and stored at −80 °C. Serial horizontal sections were cut at 20 μm using a Leica CM1850 cryostat at −18 °C. Selected sections were thawed and mounted onto Superfrost/Plus slides. The sections were rinsed in PBS and then blocked in 10% normal donkey serum in PBS for 60 min. The sections were incubated with rabbit anti-TRPA1 antibody (1:100, Santa Cruz, Dallas, TX, USA) overnight at 4 °C and FITC donkey anti-rabbit IgG (1:100, Jackson Immunoresearch Laboratories Inc., West Grove, PA, USA). Images were captured by a fluorescence microscope.

### 4.5. Total RNA Extraction and RT-PCR

Total RNA was extracted by Trizol reagent (Invitrogen Co., Carlsbad, CA, USA) from nucleus of the solitary tract (NTS) and dorsal root ganglion (DRG). cDNAs were prepared by reverse transcription. PCR was performed using a standard approach and PCR green master mix (Promega Corporation, Madison, WI, USA). The PCR products were electrophoresed in 1.5% agarose gel with ethidium bromide in Tris/Borate/EDTA buffer. The gel was scanned using Versa Doc imaging system (Bio-Rad, Hercules, CA, USA) and the blot band density was quantified by Quantity One (Bio-Rad, Hercules, CA, USA).

### 4.6. Data Analysis

All data are shown as means ± SEM. Significance is tested using unpaired Student’s *t*-test, and the data were considered significant at *p* < 0.05. For analysis of synaptic currents, Kolmogorov-Smirnov (KS) test was used to compare the cumulative probability plots for inter-event intervals and amplitude between various treatment groups. Data are represented as means ± SE and expressed as percentage of control, which is scaled to 100%.

The spontaneous/miniature postsynaptic currents (s/mPSCs) were analyzed off-line using MiniAnalysis 6.0.3 (Synaptosoft Inc., Fort Lee, NJ, USA) and the threshold for event detection (usually 10 pA) was at least three times baseline noise levels.

All the chemicals used in this study were obtained from Sigma (St. Louis, MO, USA).
